# Screening, brief intervention, and referral to treatment for alcohol and other drug use among adolescents: evaluation of a pediatric residency curriculum

**DOI:** 10.1186/1940-0640-7-S1-A98

**Published:** 2012-10-09

**Authors:** Sheryl Ryan, Shara Martel, Michael Pantalon, Steve Martino, Jeanette Tetrault, Stephen Thung, Steven Bernstein, Peggy Auinger, Gail D’Onofrio

**Affiliations:** 1 Department of Adolescent Medicine, Yale University School of Medicine, New Haven, CT, USA; 2 Department of Emergency Medicine, Yale University School of Medicine, New Haven, CT, USA; 3 Department of Psychiatry, Yale University School of Medicine, New Haven, CT, USA; 4 Department of General Internal Medicine, Yale University School of Medicine, New Haven, CT, USA; 5 Department of Obstetrics, Gynecology, and Reproductive Medicine, Yale University School of Medicine, New Haven, CT, USA; 6 Department of Neurology, University of Rochester Medical Center, Rochester, NY, USA

## 

Alcohol and other drug use and misuse are increasing in pediatric populations. As part of a US Substance Abuse and Mental Health Services Administration resident training grant, we sought to demonstrate the feasibility and effectiveness of initiating screening, brief intervention, and referral to treatment (SBIRT) in a pediatric residency program. We evaluated the efficacy of a training program for all second- and third-year pediatric and/or medicine/pediatric residents in an adolescent medicine rotation located in an urban teaching hospital. Main outcome measures were pre-/post-training knowledge scores, performance of the Brief Negotiated Interview (BNI) as measured by the BNI adherence scale in pre-/post-training standardized patient encounters (SPE), training satisfaction, and tracking of BNI performance. Thirty-four residents were trained (30 in pediatrics and four in medicine/pediatric programs). The mean age of participants was 28 years (range, 25-35 years); 26 (76%) were women. Fifty percent reported 0-5 hours of didactic training in medical school and residency. Thirty-five percent reported that they never had formal or informal teaching regarding alcohol and drug problems in their residency. There was a significant improvement in knowledge scores pre-/post-training (20.5 versus 23.4, p < 0.001) and a significant improvement in BNI adherence scores during SPE (3.1 versus 8.4, p < 0.001). Residents were very satisfied with their training, reporting a score of 1.6 on a scale of one to five (one = very satisfied, five = very dissatisfied). Integrating an SBIRT curriculum into a pediatric residency program is feasible and effective in increasing residents’ knowledge and skills in performing screening and brief interventions among adolescents and young adults.

